# SoyTEdb: a comprehensive database of transposable elements in the soybean genome

**DOI:** 10.1186/1471-2164-11-113

**Published:** 2010-02-17

**Authors:** Jianchang Du, David Grant, Zhixi Tian, Rex T Nelson, Liucun Zhu, Randy C Shoemaker, Jianxin Ma

**Affiliations:** 1 Department of Agronomy, Purdue University, West Lafayette, IN 47907, USA; 2 US Department of Agriculture-Agricultural Research Service, Corn Insect and Crop Genetics Research Unit, Ames, Iowa 50011, USA

## Abstract

**Background:**

Transposable elements are the most abundant components of all characterized genomes of higher eukaryotes. It has been documented that these elements not only contribute to the shaping and reshaping of their host genomes, but also play significant roles in regulating gene expression, altering gene function, and creating new genes. Thus, complete identification of transposable elements in sequenced genomes and construction of comprehensive transposable element databases are essential for accurate annotation of genes and other genomic components, for investigation of potential functional interaction between transposable elements and genes, and for study of genome evolution. The recent availability of the soybean genome sequence has provided an unprecedented opportunity for discovery, and structural and functional characterization of transposable elements in this economically important legume crop.

**Description:**

Using a combination of structure-based and homology-based approaches, a total of 32,552 retrotransposons (Class I) and 6,029 DNA transposons (Class II) with clear boundaries and insertion sites were structurally annotated and clearly categorized, and a soybean transposable element database, SoyTEdb, was established. These transposable elements have been anchored in and integrated with the soybean physical map and genetic map, and are browsable and visualizable at any scale along the 20 soybean chromosomes, along with predicted genes and other sequence annotations. BLAST search and other infrastracture tools were implemented to facilitate annotation of transposable elements or fragments from soybean and other related legume species. The majority (> 95%) of these elements (particularly a few hundred low-copy-number families) are first described in this study.

**Conclusion:**

SoyTEdb provides resources and information related to transposable elements in the soybean genome, representing the most comprehensive and the largest manually curated transposable element database for any individual plant genome completely sequenced to date. Transposable elements previously identified in legumes, the third largest family of flowering plants, are relatively scarce. Thus this database will facilitate structural, evolutionary, functional, and epigenetic analyses of transposable elements in soybean and other legume species.

## Background

Transposable elements (TEs) are the most abundant genomic components in flowering plants. For example, approximately 40% of the rice genome [1] and 80% of the maize genome is occupied by TEs [2]. Based on transposition mechanisms, TEs are generally classified into two types: DNA transposons and retrotransposons. DNA elements in plants are further classified into at least seven superfamilies based on their structural features and transposase similarities, whereas retrotransposons are traditionally separated into two superfamilies, the long terminal repeat (LTR)-retrotransposons and the non-LTR retrotransposons [3]. Although they are often referred to simply as 'junk DNA', more and more evidence demonstrates that TEs not only contribute to the shaping and reshaping of plant genomes and epigenomes, including centromeric regions, through their amplification, recombination, and methylation [4,5], but also play significant roles in regulating the expression of adjacent genes [6] and creating the raw material for the evolution of new genes and new genetic functions [7-9]

Identification of TEs in a species is the first step towards the understanding of their functional roles. However, precise characterization of TEs in complex genomes is not straightforward. First, many TEs, despite their abundance, have undergone intra- or inter-element unequal recombination [10,11], or accumulation of small deletions by illegitimate recombination [10,11], and thus are structurally incomplete. Second, many TEs are organized in nested patterns [12] or in chimerical structures [7], which hamper the application of programs for automated annotation of such elements. Finally, numerous elements belonging to low-copy or even single-copy number families are highly diverged within or across species, and thus are less likely to be identified by comparison with limited numbers of previously characterized elements belonging to the same families. Therefore, it remains challenging to identify and characterize the various families of TEs, especially new and low-copy number elements, in plant genomes. These TEs, as shown in rice, are apt to be mis-annotated as genes or affect the prediction of gene structures in which they reside or flank [13]. Hence, the full characterization of TEs is a critical step towards the accurate annotation of genes in a sequenced complex genome and for the investigation of interactions between TEs and genes. To this end, RetrOryza, a manually curated database of the rice LTR-retrotransposons was constructed [14]. The authors characterized many low-copy families of LTR-retrotransposons that were not collected in either Repbase [15] or the TIGR plant repeat database [16], two repeat databases that contain TEs (primarily TE fragments) from multiple plant species. In addition, manual identification and detailed analyses of DNA transposons, such as *Pack-MULEs *in rice [7] and *Helitrons *in maize [17], have been performed at the whole or nearly whole genome level, highlighting the essentiality and significance of careful characterization of TEs in individual organisms.

Soybean (*Glycine max*, 2n = 40) is the most valuable legume crop in the world, with numerous nutritional and industrial uses. Previous studies demonstrated that the soybean genome has undergone multiple whole genome level duplications [18], thus making it one of the most complex plant genomes investigated to date. Because of the economic significance of soybean, its genome has been recently sequenced and assembled by the combination of the whole-genome-shotgun (WGS) sequencing and the integration of physical and genetic maps [19]. The present pseudomolecules (Glyma1.01) of the soybean genome comprise 975 Mb of DNA that is assembled and mapped in the 20 chromosomes [19]. To facilitate the gene and genome annotation, and to better understand the organization, structure and evolution of the soybean genome, we carried out the characterization of all families of TEs in this genome, constructed a comprehensive database of soybean TEs, among which only < 5% were previously identified [20-24]. We implemented web-based sequence browsing, visualization, and comparison tools to facilitate the annotation of TEs or TE fragments in genomic sequences from soybean and other closely related legume species. In addition, the resource and tools allow users to study potential gene-TE interaction, TE-mediated gene creation, and TE-mediated evolution of duplicated regions of soybean, to identify active TEs for functional genomics, to develop TE-based molecular markers for applied studies, and to address other relevant biological questions.

## Construction and content

A combination of structure-based and homology-based approaches was employed to identify TEs in the 975 Mb of genomic sequence, but the precedures and programs used for different classes or superfamilies of TEs varied. LTR-retrotransposons were characterized by the methods previously described [25]. Non-LTR-retrotransposons, such as LINES, *Helitrons*, and other DNA transposons were identified following the protocol provided by Holligan et al [26]. More than a dozen custom perl scripts were written to facilitate the data mining and analyses. Detailed manual inspection was conducted to confirm each predicted element and to define its structure and boundaries. LTR retrotransposons were classified into different families based on the criteria proposed by Wicker et al. [3], while other elements were classified into superfamilies as previously described [26]. Only elements with clearly defined boundaries were deposited in the database.

Using the approaches above, we identified 32,370 LTR-retrotransposons, including 14,106 intact elements and 18,264 solo LTRs. These elements are classified into 510 distinct families, among which 353 were categorized into Gypsy-like families, and 157 families were assigned as Copia-like families on the basis of the order of protein coding domains [27] and/or sequence similarity. Of these families, 22 were previously described, and one of them (SIRE family) was collected in the TIGR plant repeat database to date [16]. A total of 182 LINEs with clearly defined target site duplications (TSDs) were identified, which are categorized into five distinct families. Overall, the 32,552 class I elements and numerous fragments defined by RepeatMasker [28] make up 42% of the soybean genome. In addition to the class I elements, 6,029 DNA transposons were identified, including nine Tc1-Mariners, 90 PIF-Harbingers, 65 hATs, 2,373 Mutators, 65 CACTAs, 12 PONGs and 82 Helitrons. These manually curated intact elements and fragments defined by RepeatMasker account for 16% of the soybean genome. None of these class II elements from soybean were previously collected in either Repbase or the TIGR plant repeat database. The elements identified and deposited in SoyTEdb are summarized in Table 1.

**Table 1:** Transposable elements with clear boundaries and signatures of insertion sites identified and collected in SoyTEdb

Classification	Copy numbers
Class I: Retrotransposon	32,552
LTR-Retrotransposon	32,370
*Ty1/copia*	13,318
Intact element	4,913
Solo LTR	8,405
*Ty3/gypsy*	19,052
Intact element	9,193
Solo LTR	9,859
non-LTR Retrotransposon	182
LINE	182
Class II: DNA Transposon	6,029
Subclass I:	5,947
*Tc1/Mariner*	9
*hAT*	65
*Mutator*	2,373
*PIF/Harbinger*	90
*Pong*	12
*CACTA*	65
*MITE*	3,333
*Tourist*	1,575
*Stowaway*	1,758
Subclass II:	82
*Helitron*	82
Total	38,581

## Utility

**Figure 1:**
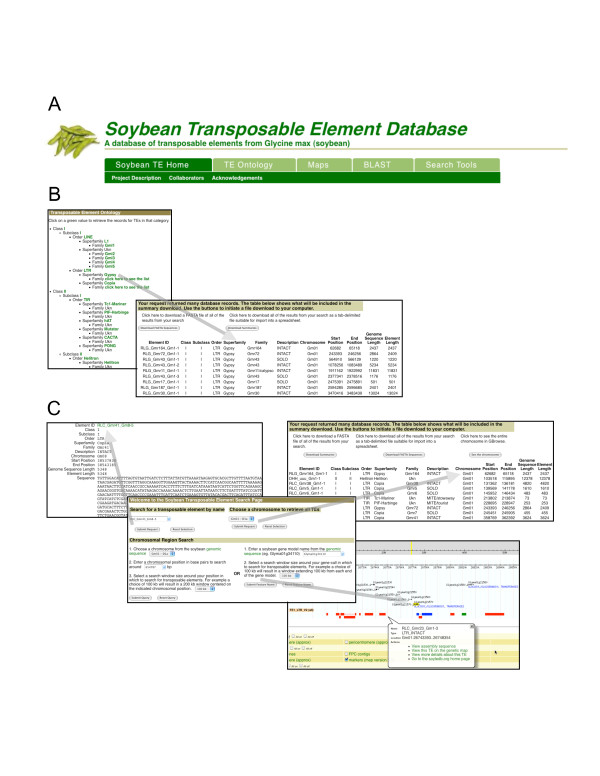
**Data for individual or specific subsets of the TEs can be retrieved using several search criteria**. A. tab bars for navigation in SoyTEdb, B. a summary of *Gypsy*-like TEs identified, and C. illustration of TEs and their organization surrounding a gene.

The SoyTEdb web interface is organized into functional sections. Each of the main navigation tabs (Figure 1A) provides a specific capability for retrieving information of TEs from the database or viewing the TEs in the context of either the genetic or genome sequence maps.

### Sorting TEs in an ontological category

TEs can be retrieved based on their ontological classification. A graphical representation of the ontology is presented (Figure 1B). Clicking on a node retrieves all of the TEs in the ontology hierarchy from that node downwards. Because the list of TEs will typically be very large, a summary of the search results is shown with the entire results available for download in either tab-delimited or FASTA format.

### Finding TEs around genes

**Figure 2:**
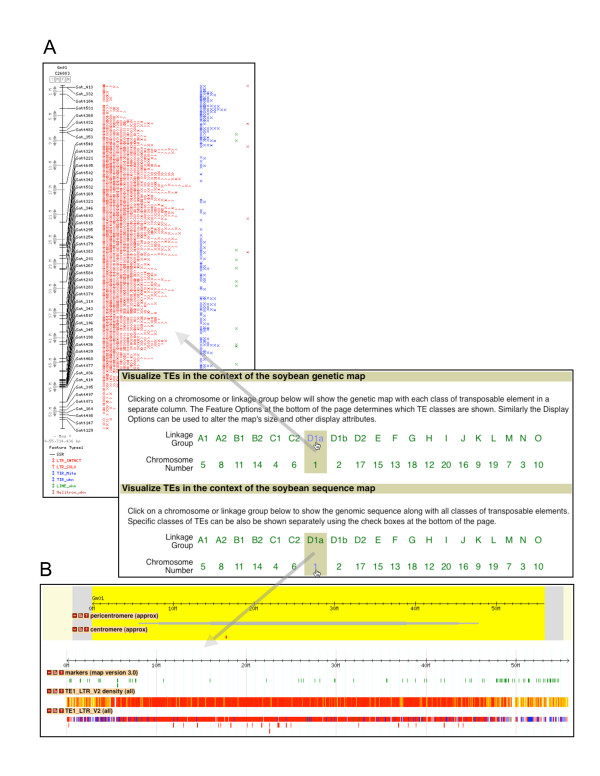
**Visulization of TEs in the context of genetic map and genome sequence**. A. The distribution of TEs in the context of the genetic map of chromosome Gm01, and B. the distribution of TEs in the context of the genomic sequence of chromosome Gm01.

A list of the TEs for an entire chromosome or in a user defined window around either a chromosomal position or a gene model can be generated (Figure 2C). Each TE is annotated with chromosome and start/stop position, the complete ontology classification and a short description of the TE's structure. These data can be downloaded in a tab-delimited or FASTA format which includes the sequences of the TEs. This function can help users to identify TEs that surround the genes of interests, and study the interaction between TEs and genes.

### Visualizing TEs in the context of genetic map and genome sequence

The soybean TEs can be viewed in the context of either the composite soybean genetic map or the Williams 82 genomic sequence (Figure 2). These views are accomplished using the CMap and GBrowse components of The GMOD Project [29]. The genetic map view is useful for obtaining an overview of the TE distribution and genetic marker distribution for a chromosomal region or an entire chromosome (Figure 2A). As TEs are largely enriched in the recombination-low heterochromatic regions or other gene-poor regions, where few genetic markers are generally mapped, the integration of TE distribution and genetic map can help users to develop unique repeat-junction markers [30] that can be used for construction of finer genetic map or mapping of genes of interest. The sequence map view allows users to zoom into a region of the chromosome and see the TEs relative to the other sequence annotations (gene models, transcripts, etc.) (Figures 1C and 2B), and thus allows users to identify TEs that may alter the structures and/or regulate the expression of genes. Nested TEs are indicated in the sequence map displays using the familiar box & line glyphs (Figure 1C). The genetic and sequence displays are interconnected via contextual menus, which also allow a quick retrieval of all of the information available for a specific TE.

### Searching sequence similarity using BLAST

Because the structural variation and distribution patterns of TEs vary among classes and among families, a single annotation pipeline cannot satisfy all users with different interests. Thus, we did not intend to develop new tools or to integrate tools currently available (except for BLAST) for sequence comparison, editing and/or assembly in our database infrastructure. However, the SoyTEdb web provides the canonical web BLAST interface, which allows users handy and quick comparison of their sequences with the soybean TEs deposited in SoyTEdb.

## Discussion

We established SoyTEdb under the infrastructure of SoyBase and the Soybean Breeder's Toolbox [31]. As such, SoyTEdb represents the only TE database with components of integration with a genetic map and physical map, with annotation tools, annotations of other DNA components, as well as nearly 20 years of quantatitive trait locus (QTL) analyses of agronomically important genes. SoyBase and the Soybean Breeder's Toolbox were described in the "National Plant Genome Initiative: 2009-2013" [32] as databases that bridge genomics and application for crop improvement. Thus SoyTEdb can be used for both basic research and applied studies, such as marker development for mapping agronomically important genes. It is also easily used for both intra- and inter-specific comparison of transposable elements at whole genome levels.

In light of recent discoveries made from detailed analysis of TEs in plants, such as rice and maize [7,8], the importance of creating a complete TE database from an individual genome can be substantial. Although the TIGR plant repeat database is currently available, it only collected approximately 4,000 TEs, of which, many were fragments and very few were manually inspected. In addition, the majority of TEs collected in the TIGR database are from grasses, and very few were identified in legumes, the third largest family of flowering plants. For example, only 23, eight, and zero TEs or fragments were collected from soybean, *Lotus*, and *Medicago*, respectively. It thus is not surprising that this database was rarely used for annotation of even the rice genome. By contrast, RetrOryza, a manually cruated rice LTR-retrotransposon database, despite its incompleteness [33], has served as an essential resource for the reannotation of the rice genome [34]. Thus, manual annotation of a complete set of TEs are desirable for any genome sequencing projects and research community.

## Conclusion

We have generated a comprehensive database of transposable elements, of which, ~95% were first identified in this study and ~5% were identified in previous studies (19-23). This database has been used in the soybean genome annotation pipeline to facilitate accurate annotation of the soybean genes. SoyTEdb will be valuable as the legume community undertakes the structural and functional characterization of TEs and their interaction with genes in soybean and related legume species. In addition, the availability of the complete set of TEs from a complex dicot genome allows evolutionary and comparative analyses of TEs between dicot and monocot species at the whole genome level.

## Future perspectives

Future SoyTEdb development includes the integration of TE data from *Glycine soja*, other *Glycine *species, and common bean, whose genomes will be completely or partially sequenced [SoyMapII project supported by the US NSF Plant Genome Research Program Grant # DBI-0822258; Common Bean Sequencing Project to be supported by the USDA Agriculture and Food Research Initiative (Jackson, pers. Comm.)]. In addition, genes captured by TEs and TEs that carry gene fragments in soybean and these relatives will be identified, classified and integrated into the database in the context of the comparative genome maps of multiple species.

## Availability and requirements

All TEs or subsets of TEs can be downloaded from the SoyTEdb website http://www.soytedb.org, which is publicly accessible. These data are freely available without any restrictions to use by non-academics.

## Abbreviations

LINE: Long interspersed repetitive element; LTR: Long terminal repeat; SoyTEdb: Soybean Transposable Element Database; TE: Transposable element; TSD: Target site duplication; WGS: whole genome shotgun sequencing.

## Authors' contributions

JD, ZT and LZ identified transposable elements. DG and RTN constructed the web-based database and helped to draft the manuscript. RCS and JM conceived of the study, participated in its design and corordination, and drafted the manuscript, and served as principle investigators of the project. All authors read and approved the final manuscript.

## References

[B1] International Rice Genome Sequencing Project The map-based sequence of the rice genome Nature 2005 436 793 800 10.1038/nature03895 16100779

[B2] Meyers BC Tingey SV Morgante M Abundance, distribution, and transcriptional activity of repetitive elements in the maize genome Genome Res 2001 11 1660 1676 10.1101/gr.188201 11591643 PMC311155

[B3] Wicker T Sabot F Hua-Van A Bennetzen JL Capy P Chalhoub B Flavell A Leroy P Morgante M Panaud O A unified classification system for eukaryotic transposable elements Nat Rev Genet 2007 8 973 982 10.1038/nrg2165 17984973

[B4] Ma J Bennetzen JL Recombination, rearrangement, reshuffling, and divergence in a centromeric region of rice Proc Natl Acad Sci USA 2006 103 383 388 10.1073/pnas.0509810102 16381819 PMC1326179

[B5] Zhang W Lee HR Koo DH Jiang J Epigenetic modification of centromeric chromatin: hypomethylation of DNA sequences in the CENH3-associated chromatin in *Arabidopsis thaliana *and maize Plant Cell 2008 20 25 34 10.1105/tpc.107.057083 18239133 PMC2254920

[B6] Kashkush K Feldman M Levy AA Transcriptional activation of retrotransposons alters the expression of adjacent genes in wheat Nat Genet 2003 33 102 106 10.1038/ng1063 12483211

[B7] Jiang N Bao Z Zhang X Eddy SR Wessler SR Pack-MULE transposable elements mediate gene evolution in plants Nature 2004 431 569 573 10.1038/nature02953 15457261

[B8] Morgante M Brunner S Pea G Fengler K Zuccolo A Rafalski A Gene duplication and exon shuffling by helitron-like transposons generate intraspecies diversity in maize Nat Genet 2005 37 997 1002 10.1038/ng1615 16056225

[B9] Bennetzen JL Transposable elements, gene creation and genome rearrangement in flowering plants Curr Opin Genet Dev 2005 15 621 627 10.1016/j.gde.2005.09.010 16219458

[B10] Devos KM Brown JK Bennetzen JL Genome size reduction through illegitimate recombination counteracts genome expansion in *Arabidopsis* Genome Res 2002 12 1075 1079 10.1101/gr.132102 12097344 PMC186626

[B11] Ma J Devos KM Bennetzen JL Analyses of LTR-retrotransposon structures reveal recent and rapid genomic DNA loss in rice Genome Res 2004 14 860 869 10.1101/gr.1466204 15078861 PMC479113

[B12] SanMiguel P Tikhonov A Jin YK Motchoulskaia N Zakharov D Melake-Berhan A Springer PS Edwards KJ Lee M Avramova Z Nested retrotransposons in the intergenic regions of the maize genome Science 1996 274 765 768 10.1126/science.274.5288.765 8864112

[B13] Bennetzen JL Coleman C Liu R Ma J Ramakrishna W Consistent over-estimation of gene number in complex plant genomes Curr Opin Plant Biol 2004 7 732 736 10.1016/j.pbi.2004.09.003 15491923

[B14] Chaparro C Guyot R Zuccolo A Piegu B Panaud O RetrOryza: a database of the rice LTR-retrotransposons Nucleic Acids Res 2007 35 D66 70 10.1093/nar/gkl780 17071960 PMC1635335

[B15] Jurka J Kapitonov VV Pavlicek A Klonowski P Kohany O Walichiewicz J Repbase Update, a database of eukaryotic repetitive elements Cytogenet Genome Res 2005 110 462 467 10.1159/000084979 16093699

[B16] Ouyang S Buell CR The TIGR Plant Repeat Databases: a collective resource for the identification of repetitive sequences in plants Nucleic Acids Res 2004 32 D360 363 10.1093/nar/gkh099 14681434 PMC308833

[B17] Yang L Bennetzen JL Structure-based discovery and description of plant and animal Helitrons Proc Natl Acad Sci USA 2009 106 12832 12837 10.1073/pnas.0905563106 19622734 PMC2722332

[B18] Shoemaker RC Schlueter J Doyle JJ Paleopolyploidy and gene duplication in soybean and other legumes Curr Opin Plant Biol 2006 9 104 109 10.1016/j.pbi.2006.01.007 16458041

[B19] Schmutz J Cannon S Schlueter J Ma J Hyten D Cregan P Mitros T Nelson W Goodstein D Thelen JJ Genome sequence of the palaeopolyploid soybean Nature 2010 463 178 183 10.1038/nature08670 20075913

[B20] Jarvik T Lark KG Characterization of Soymar1, a mariner element in soybean Genetics 1998 149 1569 1574 9649543 10.1093/genetics/149.3.1569 PMC1460244

[B21] Laten HM Majumdar A Gaucher EA *SIRE-1*, a *copia/Ty1*-like retroelement from soybean, encodes a retroviral envelope-like protein Proc Natl Acad Sci USA 1998 95 6897 6902 10.1073/pnas.95.12.6897 9618510 PMC22677

[B22] Yano ST Panbehi B Das A Laten HM Diaspora, a large family of *Ty3-gypsy *retrotransposons in *Glycine max*, is an envelope-less member of an endogenous plant retrovirus lineage BMC Evol Biol 2005 5 30 10.1186/1471-2148-5-30 15876351 PMC1142308

[B23] Wawrzynski A Ashfield T Chen NW Mammadov J Nguyen A Podicheti R Cannon SB Thareau V Ameline-Torregrosa C Cannon E Replication of nonautonomous retroelements in soybean appears to be both recent and common Plant Physiol 2008 148 1760 1771 10.1104/pp.108.127910 18952860 PMC2593652

[B24] Innes RW Ameline-Torregrosa C Ashfield T Cannon E Cannon SB Chacko B Chen NW Couloux A Dalwani A Denny R Differential accumulation of retroelements and diversification of NB-LRR disease resistance genes in duplicated regions following polyploidy in the ancestor of soybean Plant Physiol 2009 148 1740 1759 10.1104/pp.108.127902 PMC2593655 18842825

[B25] Ma J Jackson SA Retrotransposon accumulation and satellite amplification mediated by segmental duplication facilitate centromere expansion in rice Genome Res 2006 16 251 259 10.1101/gr.4583106 16354755 PMC1361721

[B26] Holligan D Zhang X Jiang N Pritham EJ Wessler SR The transposable element landscape of the model legume *Lotus japonicus* Genetics 2006 174 2215 2228 10.1534/genetics.106.062752 17028332 PMC1698628

[B27] Kumar A Bennetzen JL Plant retrotransposons Annu Rev Genet 1999 33 479 532 10.1146/annurev.genet.33.1.479 10690416

[B28] Smit A Hubley R Green P RepeatMasker http://www.repeatmasker.org

[B29] GMOD, the Generic Model Organism Database project http://gmod.org

[B30] Devos KM Ma J Pontaroli AC Pratt LH Bennetzen JL Analysis and mapping of randomly chosen bacterial artificial chromosome clones from hexaploid bread wheat Proc Natl Acad Sci USA 2005 102 19243 19248 10.1073/pnas.0509473102 16357197 PMC1323192

[B31] Grant D Nelson RT Cannon SB Shoemaker RC SoyBase, the USDA-ARS soybean genetics and genomics database Nucleic Acids Res 2010 38 Database D843 846 10.1093/nar/gkp798 20008513 PMC2808871

[B32] The "National Plant Genome Initiative: 2009-2013" 2009 http://www.whitehouse.gov/administration/eop/ostp/nstc

[B33] Tian Z Rizzon C Du J Liu Z Bennetzen JL Jackson SA Gaut B Ma J Do genetic recombination and gene density shape the pattern of DNA elimination in rice LTR-retrotransposons? Genome Res 2009 11 2221 2230 10.1101/gr.083899.108 PMC2792168 19789376

[B34] Rice Annotation Project Tanaka T Antonio BA Kikuchi S Matsumoto T Nagamura Y Numa H Sakai H Wu J Itoh T Sasaki T Aono R The Rice Annotation Project Database (RAP-DB): 2008 update Nucleic Acids Res 2008 36 Database D1028 1033 18089549 10.1093/nar/gkm978 PMC2238920

